# Risk assessment and prediction of TD incidence in psychiatric patients taking concomitant antipsychotics: a retrospective data analysis

**DOI:** 10.1186/s12883-019-1385-4

**Published:** 2019-07-20

**Authors:** Oscar Patterson-Lomba, Rajeev Ayyagari, Benjamin Carroll

**Affiliations:** 1 Analysis Group, Inc., 111 Huntington Avenue, Boston, MA 02199 USA; 2 Teva Pharmaceuticals, 41 Moores Rd, Malvern, PA 19355 USA

**Keywords:** Tardive dyskinesia, Risk factors, Psychiatric patients, antipsychotics, Prediction model, Least absolute shrinkage and selection operator

## Abstract

**Background:**

Tardive dyskinesia (TD) is a serious, often irreversible movement disorder caused by prolonged exposure to antipsychotics; identifying patients at risk for TD is critical to preventing it. Predictive models for the occurrence of TD can improve patient monitoring and inform implementation of counteractive interventions. This study aims to identify risk factors associated with TD and to develop a model using a retrospective data analysis to predict the incidence of TD among patients taking antipsychotic medications.

**Methods:**

Adult patients with schizophrenia, major depressive disorder, or bipolar disorder taking oral antipsychotics were identified in a Medicaid claims database (covering six US states from 1997 to 2016) and divided into cohorts based on whether they developed TD within 1 year after the first observed claim for antipsychotics. Patient characteristics between cohorts were compared, and univariate Cox analyses were used to identify potential TD risk factors. A cross-validated version of the least absolute shrinkage and selection operator regression method was used to develop a parsimonious multivariable Cox proportional hazards model to predict diagnosis of TD.

**Results:**

A total of 189,415 eligible patients were identified. Potential TD risk factors were identified based on the cohort analysis within a sample of 151,280 patients with at least 1 year of continuous eligibility. The prediction model had a clinically meaningful concordance of 70% and was well calibrated (*P* = 0.32 for Hosmer–Lemeshow goodness-of-fit test). Age (hazard ratio [HR] = 1.04, *P* < 0.001), diagnosis of schizophrenia (HR = 1.99, *P* < 0.001), antipsychotic dosage (up to 100 mg/day chlorpromazine equivalent; HR = 1.65, *P* < 0.01), and comorbid bipolar and related disorders (HR = 1.39, *P* < 0.01) were significantly associated with an increased risk of TD. Other potential risk factors included history of extrapyramidal symptoms (HR = 1.35), other movement disorders (parkinsonism, HR = 1.43; bradykinesia, HR = 1.44; tremors, HR = 2.12, and myoclonus, HR = 2.33), and diabetes (HR = 1.13). A modest reduction in the risk of TD was associated with the use of second-generation antipsychotics (HR = 0.85) versus first-generation drugs.

**Conclusions:**

This study identified factors associated with development of TD among patients taking antipsychotics. The prediction model described herein can enable physicians to better monitor patients at high risk for TD and recommend appropriate treatment plans to help maintain quality of life.

**Electronic supplementary material:**

The online version of this article (10.1186/s12883-019-1385-4) contains supplementary material, which is available to authorized users.

## Background

Tardive dyskinesia (TD) is a hyperkinetic, potentially irreversible movement disorder that is typically caused by prolonged exposure to antipsychotic drugs [1–6]. The clinical manifestations include abnormal movements of the face, lips, tongue, cheeks, jaws and extremities, and severity of symptoms can range from mild to disabling and potentially life-threatening [1, 2, 6–9]. Besides the physical discomfort experienced due to the disease, the involuntary, repetitive and pronounced nature of TD symptoms can exacerbate the stigmatization often already faced by patients with mental illness, leading to social alienation, behavioral disturbances and nonadherence [2, 3, 5, 10].

Motor side effects have been reported in as many as 40% of patients receiving antipsychotics; thus, elucidation of both modifiable and nonmodifiable risk factors for TD susceptibility remains a research priority [4, 11]. An increased risk for developing TD has been associated with older age, female sex, underlying mental disorders, history of extrapyramidal symptoms (EPS), diabetes, and higher antipsychotic dose and longer duration of exposure to antipsychotics [7, 12–15]. Several studies also suggest a higher risk for TD incidence with the use of first-generation (typical) compared with second-generation (atypical) antipsychotic drugs [4, 7, 16]. A 2008 review reported annual TD incidence rates in adults of 3.0% with second-generation antipsychotics versus 7.7% with first-generation antipsychotics [17]. In contrast, results from various groups show similar frequencies of TD occurrence regardless of the class of antipsychotic drug treatment, and that movement disorders associated with newer antipsychotic drugs are not clinically negligible when taking into consideration methodological differences (e.g. study population, clinical setting and differential diagnosis) that can potentially lead to the underestimation of incidence in patients treated with second-generation antipsychotics [18–20]. Therefore, no consensus currently exists on the exact epidemiology of TD [17, 19, 20].

Prevention and treatment of TD continue to pose significant challenges to clinicians [2, 20]. First, detection of TD onset can be delayed due to inconsistent clinical presentations, significant variability in developmental timelines, masking of symptoms by the very drugs that cause TD, and misclassification of motor symptoms as medication-induced side effects [1, 3, 5, 9, 19, 21]. Even after a definitive diagnosis is made, the lowering or cessation of treatment with causative drugs may be contraindicated due to aggravation of psychosis and other symptoms of underlying comorbidities in TD patients [2, 6, 19]. Furthermore, considering that TD is sometimes irreversible, early diagnosis or withdrawal from antipsychotic therapy may confer only partially ameliorative benefits [3, 21].

Although novel drugs for treatment have been recently approved for TD [22], the best management strategy should include better monitoring and implementation of risk-stratified prophylactic measures, such as the modification of treatment plans for patients at risk of developing TD [2, 4, 23]. Further investigation for potential risk factors to identify “true predictors” of disease is warranted to accurately identify high-risk populations [3, 11]. In the current study, we developed and validated a predictive model assessing the combined effect of clinical characteristics on TD risk, which, to our knowledge, is the first of its kind for US populations. The resulting prediction model has the potential to guide decision-making regarding treatment and follow-up management.

## Methods

### Study objective and data sources

A retrospective cohort study was conducted to identify risk factors and develop a model to predict the incidence of TD among psychiatric patients taking antipsychotic medication. Medicaid claims data from a database that represented a sample of the total Medicaid beneficiaries in the US from six states (Iowa, Kansas, Missouri, New Jersey, Mississippi and Wisconsin) were extracted. The claims data included services provided (for most states) from 1997 through the first quarter of 2016. Complete medical claims (e.g. procedures, paid amounts and diagnoses), pharmaceutical claims, enrollment history, and patient demographics were available for analysis from the Medicaid records. The most recent 6 years of data (varies by state) were used for this analysis.

### Patient selection

Patients with schizophrenia, major depressive disorder, or bipolar disorder, who were taking antipsychotic medications and who also satisfied the following eligibility criteria were selected from Medicaid claims database (the most recent 6 years of data of each state): at least two diagnoses for schizophrenia (International Classification of Diseases, Ninth Revision, Clinical Modification [ICD-9-CM] codes: 295.xx; International Classification of Diseases, Tenth Revision, Clinical Modification [ICD-10-CM] codes: F20.x), major depressive disorder (296.2, 296.3; F32.xx, F33.xx), or bipolar disorder (296.0, 296.1, 296.4–296.8; F31.x); at least one oral antipsychotic fill (see Additional file 1 ICD-9-CM and ICD-10-CM Codes for Selected Comorbidities and GPIs for Antipsychotics) after first observed diagnosis for the underlying disorder and before any observed TD diagnosis (333.81, 333.82, 333.85; G24.01, G24.4, G24.5); ≥18 years of age at index date (date of the first observed antipsychotic fill); a baseline period of continuous eligibility for ≥6 months before the index date; and cases with daily doses that are not missing nor daily dose outliers (i.e. daily dose > 1,200 mg/day chlorpromazine equivalent [24–26]). Patients from New Jersey who turned 65 after 2012 were dual-eligible for Medicare and Medicaid and thus excluded from the study to eliminate the possibility of incomplete capture of their drug claim information. The study period was defined from the index date to the end of eligibility or end of data. There was no minimum time requirement for post-index eligibility.

### Patients characteristics and study variables

The following patient information was collected: demographics (age, gender, state, and health plan); disease duration (from first observed diagnosis of schizophrenia, or depression, or bipolar disorder to index date); index antipsychotic treatment by class (i.e. the treatment the patients were treated with on the index date, which can be a first-generation antipsychotic, a second-generation antipsychotic, both or none); comorbidity profile, including psychiatric comorbidities, Charlson Comorbidity Index (CCI) score (a method of categorizing comorbidities based on ICD codes, where each comorbidity category has a weight associated to its risk of mortality or resource use, and the sum of the weights results in a single score) [27, 28], brain damage, diabetes, dementia, parkinsonism, and other selected comorbidities; EPS other than TD, e.g. akathisia, parkinsonism, dystonia, and tremors; cognitive disabilities such as Down’s syndrome, autism, dyslexia and other scholastic disorders; traumatic brain injury; smoking history and alcohol abuse; diabetes; and duration of follow-up (see Additional file 1 ICD-9-CM and ICD-10 CM Codes for Selected Comorbidities and GPIs for Antipsychotics). The main outcome was time to TD diagnosis after index date.

### Risk factor identification

Patients with at least 1 year of continuous eligibility after their index date were divided into two cohorts: those who developed TD within 1 year, and those who did not develop TD within 1 year. Patient characteristics were then compared between the two cohorts to identify potential risk factors for TD. Means and standard deviations were summarized for continuous variables, whereas frequencies and percentages were summarized for categorical variables. Statistical comparisons were conducted using Wilcoxon rank-sum tests for continuous variables, McNemar’s test for dichotomous variables, and chi-squared tests for categorical variables. For mutually exclusive categorical variables with more than two categories, the statistical comparisons were conducted using Bowker’s test for symmetry.

Univariate Cox regression models were also used to assess the association of each patient baseline characteristic with the risk of TD diagnosis among all selected patients. Time to event was estimated as the period from index date to the first TD claim. Patients without the event of interest during the study period were censored at the end of their follow-up period.

### Development and validation of predictive model

Data were separated randomly into a modeling set (two-thirds of the data), used to develop and parametrize the prediction model, and a validation set (one-third of the data), used to test out-of-sample performance of the prediction model.

A multivariable Cox proportional hazard model was developed using the modeling set to predict the time to TD diagnosis in patients taking antipsychotics at a given time point after the index date. The variables in the model included the aforementioned patient characteristics as potential predictors based on the univariate Cox models and “TD” versus “no TD” cohort comparisons. Based on the non-linear empirical relationships between the probability of TD diagnosis with age and dose, predictors used also included transformed dose and age variables. Covariates in the model (before selection) were: age at index date; sex; index diagnosis; type of index antipsychotic; history and number of EPS; dose, transformed dose (as a continuous effect for doses up to 100 mg/day of chlorpromazine equivalents, and as a continuous effect for doses larger than 100 mg/day of chlorpromazine equivalents); CCI; comorbid movement disorders, including parkinsonism, akathisia, bradykinesia, tremors, and myoclonus; comorbid psychiatric disorders, including anxiety disorders, depressive disorders, bipolar and related disorders; and other factors, including brain damage, dementia, diabetes, and alcohol history. Interactions between underlying type of mental disorder and treatment patterns, or between sex and age were also included in the model. The least absolute shrinkage and selection operator (LASSO) regression method was used to simultaneously estimate the model and identify the patient characteristics that better predicted TD. The model was selected to minimize a cross-validated prediction error, which helped to avoid overfitting and to enhance the interpretability of the model. A Cox regression was then performed with only the selected covariates from the LASSO regression to obtain HR estimates and the corresponding *P* value associated with each of the model variables. Risk factors for TD were then characterized based on effect size and significance.

Predictive performance was assessed in the validation set by: 1) model discrimination or concordance, which is the ability of the model to distinguish between low and high-risk patients, quantified by the C statistics (C = 0.5 is random prediction, and C = 1 is perfect prediction); and 2) model calibration, which determines the agreement between the observed and predicted risk of TD at any given time after the index date, quantified by the Hosmer–Lemeshow goodness-of-fit test (*P* > 0.05 suggests a good fit to the data, i.e. good calibration). The Breslow estimator of the baseline hazard was combined with the HRs to obtain predicted risks of TD for each patient at 2 years after the index date.

## Results

### Baseline characteristics by diagnosis

A total of 189,415 patients met the inclusion criteria in the Medicaid claims database used (see Additional file 2 Sample Selection Flow Chart). Patient characteristics and treatment history are summarized for all patients and by initial psychiatric diagnosis in Table 1. Briefly, the mean age of all patients was 42.8 years (bipolar, 39.6 years; depressive disorder, 43.9 years; schizophrenia, 45.4 years); 38.1% of all patients were men (bipolar, 32.6%; depressive disorder, 28.7%; schizophrenia, 56.9%); the overall average daily dose of antipsychotic medication was 220 mg chlorpromazine equivalent (bipolar, 211 mg; depressive disorder, 157 mg; schizophrenia, 309 mg). The vast majority of all patients (86.7%) were prescribed a second-generation antipsychotic at index date (bipolar, 91.3%; depressive disorder, 88.8%; schizophrenia, 81.4%). The comorbidity profiles were different among the three diagnostic groups; patients with schizophrenia showed the lowest CCI scores, as well as lower rates of substance-related and addictive disorders, anxiety disorders, personality disorders, trauma- and stressor-related disorders, brain damage, and smoking history.

**Table 1:** Patient demographics and baseline characteristics by diagnosis

Patient characteristics	Total *N* = 189,415	Bipolar disorder *N* = 66,723	Depressive disorder *N* = 68,573	Schizophrenia *N* = 54,119
Demographics				
Age (years)	42.8 + 13.8	39.6 + 13.0	43.9 + 13.8	45.4 ± 13.9
Male, % (n)	38.1% (72,187)	32.6% (21,749)	28.7% (19,662)	56.9% (30,776)
HMO Plan, % (n)	20.3% (38,494)	21.2% (14,133)	20.8% (14,227)	18.7% (10,134)
State				
Iowa	8.1% (15,390)	10.8% (7,226)	7.8% (5,366)	5.2% (2,798)
Kansas	8.3% (15,698)	8.8% (5,851)	8.0% (5,469)	8.1% (4,378)
Mississippi	8.9% (16,756)	7.1% (4,741)	9.5% (6,539)	10.1% (5,476)
Missouri	38.7% (73,293)	38.9% (25,924)	43.3% (29,694)	32.7% (17,675)
New Jersey	21.5% (40,667)	19.1% (12,737)	18.5% (12,659)	28.2% (15,271)
Wisconsin	14.6% (27,611)	15.4% (10,244)	12.9% (8,846)	15.7% (8,521)
Observed disease duration (months)	7.8 ± 13.0	7.6 ± 12.8	9.2 ± 13.6	6.2 ± 12.2
Duration of follow-up (months)	38.6 ± 24.6	37.4 ± 23.8	32.6 ± 23.5	47.8 ± 24.2
Index AP use				
First generation	10.4% (19,673)	7.4% (4,958)	10.6% (7,239)	13.8% (7,476)
Multiple	2.0% (3,849)	1.2% (818)	0.7% (448)	4.8% (2,583)
Second generation	87.6% (165,893)	91.3% (60,947)	88.8% (60,886)	81.4% (44,060)
Chlorpromazine equivalent daily dose (100 mg/day)	2.2 ± 2.1	2.1 ± 1.9	1.6 ± 1.6	3.1 ± 2.4
Psychiatric comorbidities				
Substance-related and addictive disorders	23.8% (45,014)	27.1% (18,090)	26.2% (17,944)	16.6% (8,980)
Anxiety disorders	21.9% (41,438)	23.0% (15,322)	31.5% (21,603)	8.3% (4,513)
Autism	0.6% (1,149)	0.9% (614)	0.4% (258)	0.5% (277)
Bipolar and related disorders	32.6% (61,715)	77.9% (51,959)	9.8% (6,743)	5.6% (3,013)
Depressive disorders	45.2% (85,537)	24.3% (16,195)	90.2% (61,862)	13.8% (7,480)
Personality disorders	4.0% (7,526)	4.6% (3,045)	4.6% (3,168)	2.4% (1,313)
Schizophrenia spectrum disorders (excluding schizophrenia	8.2% (15,550)	6.1% (4,094)	6.6% (4,511)	12.8% (6,945)
Sleep-wake disorders	8.5% (16,024)	8.8% (5,838)	11.4% (7,845)	4.3% (2,341)
Trauma- and stress- or related disorders	10.0% (19,004)	10.8% (7,178)	14.4% (9,883)	3.6% (1,943)
Other comorbidities				
CCI	0.6 ± 1.2	0.5 ± 1.1	0.7 ± 1.4	0.4 ± 1.0
Alcohol history	7.7% (14,592)	8.2% (5,461)	8.7% (5,958)	5.9% (3,173)
Brain damage	1.0% (1,790)	0.8% (563)	1.3% (885)	0.6% (342)
Dementia	1.6% (3,080)	0.9% (624)	2.0% (1,392)	2.0% (1,064)
Diabetes	14.0% (26,600)	11.6% (7,767)	16.4% (11,215)	14.1% (7,618)
Down’s Syndrome	0.1% (200)	0.1% (67)	0.1% (84)	0.1% (49)
Dyslexia and other scholastic disorders	0.1% (254)	0.1% (68)	0.2% (123)	0.1% (63)
Smoking history	13.7% (25,927)	16.2% (10,793)	15.1% (10,337)	8.9% (4,797)
Traumatic brain injury	0.4% (673)	0.3% (207)	0.5% (317)	0.3% (149)
Extrapyramidal symptoms				
Akathisia	0.1% (248)	0.1% (94)	0.1% (90)	0.1% (64)
Bradykinesia	3.3% (6,251)	3.0% (1,973)	4.2% (2,908)	2.5% (1,370)
Dystonia	0.1% (167)	0.1% (60)	0.1% (34)	0.1% (73)
EPS (unspecified)	0.8% (1,430)	0.8% (559)	0.9% (636)	0.4% (235)
Myoclonus	0.1% (120)	0.1% (37)	0.1% (64)	0.04% (19)
Malignant neuroleptic syndrome	0.02% (46)	0.02% (13)	0.01% (4)	0.1% (29)
Parkinsonism	0.1% (141)	0.1% (34)	0.1% (31)	0.1% (76)
Drug-induced tics	0.0% (7)	0.0% (3)	0.01% (4)	0.0% (0)
Tremors	0.3% (490)	0.3% (183)	0.3% (222)	0.2% (85)
History of EPS	4.3% (8,063)	4.0% (2,663)	5.4% (3,668)	3.2% (1,732)
Number of EPS	0.1 ± 0.2	0.04 ± 0.23	0.1 ± 0.3	0.04 ± 0.21

*AP* antipsychotic, *CCI* Charlson Comorbidity Index, *EPS* extrapyramidal symptoms, *HMO* health maintenance organization, *TD* tardive dyskinesia

### Comparison of baseline characteristics by TD cohort

A sample of 151,280 patients with at least 1 year of continuous eligibility after the index date was used to identify potential risk factors of TD. A total of 381 patients developed TD within 1 year and were classified as ‘TD,’ and the remaining 150,899 patients who did not develop TD within 1 year were labeled as ‘No TD.’ Age, diagnosis of schizophrenia, use of first-generation antipsychotics, antipsychotic dose, CCI, diabetes, and incidence of EPS-related comorbidities were significantly higher at baseline in the ‘TD’ cohort than in the ‘No TD’ cohort. The characteristics that were significantly different between the two cohorts are shown in Table 2.

**Table 2:** Patient demographics and baseline characteristics by TD cohort

Patient characteristics	TD *N* = 381	No TD *N* = 150,899	*P* value
Age (years)	51.4 ± 13.2	43.3 ± 13.6	< 0.001
Index Diagnosis, %(n)			
Bipolar disorder	24.2% (92)	35.2% (53,169)	
Depressive disorder	21.8% (83)	33.3% (50,213)	< 0.001
Schizophrenia	54.1% (206)	31.5% (47,517)	
Generation of index AP			
First generation	16.8% (64)	10.5% (15,850)	
Multiple	3.4% (13)	2.3% (3,424)	< 0.001
Second generation	79.8% (304)	87.2% (131,625)	
Chlorpromazine equivalent daily dose (100 mg/day)	2.78 ± 2.29	2.29 ± 2.11	< 0.001
CCI	0.64 ± 1.18	0.53 ± 1.16	0.05
Diabetes	23.9% (91)	14.4% (21,674)	< 0.001
Bipolar and related disorders	26.0% (99)	31.9% (48,075)	< 0.05
Depressive disorders	32.6% (124)	42.6% (64,301)	< 0.001
Bradykinesia	9.7% (37)	3.3% (4,917)	< 0.001
Dystonia	0.8% (3)	0.1% (131)	< 0.001
Myoclonus	1.1% (4)	0.1% (91)	< 0.001
Parkinsonism	0.8% (3)	0.1% (119)	< 0.001
History of EPS	12.3% (47)	4.2% (6,388)	< 0.001
Number of EPS	0.14 ± 0.41	0.05 ± 0.23	< 0.001

*AP* antipsychotic, *CCI* Charlson Comorbidity Index, *EPS* extrapyramidal symptoms, *TD* tardive dyskinesia

### Identification of TD predictors using univariate Cox analyses

Univariate Cox analysis was conducted in the full sample of 189,415 patients to identify potential risk factors for TD in psychiatric patients taking concurrent antipsychotic medication (Table 3). The results suggest associative relationships between TD onset and mostly the same baseline risk factors identified by the cohort analysis described above (Table 2). According to the univariate Cox model, a significant increase in risk of TD was found to be associated with diagnosis of schizophrenia (HR = 1.96 compared with bipolar), antipsychotic dose (up to 100 mg/day of chlorpromazine, HR = 1.91), dementia (HR = 2.04), EPS-related comorbidities (number of EPS, HR = 1.91; history of EPS, HR = 2.37) and diabetes (HR = 1.52). A small but significant association was determined for CCI (HR = 1.06) and age (HR = 1.04). Compared with first-generation antipsychotics, use of second-generation antipsychotics was associated with a lower risk of TD (HR = 0.72), and so was use of multiple-generation antipsychotics (HR = 0.88). Furthermore, depressive (HR = 0.78) and bipolar-related disorders (HR = 0.84) were associated with a significant decrease in the risk of TD. Finally, other movement disorders (parkinsonism, HR = 4.29; myoclonus, HR = 4.27; tremors, HR = 3.93; and bradykinesia, HR = 2.48) were associated with significantly higher risk of TD (Table 3).

**Table 3:** Hazard ratio for risk factors using variables selected by the LASSO method

Patient characteristics	Univariate Cox analyses			Variables selected by the LASSO method		
	Hazard Ratio	95% CI	*P* value	Hazard ratio	*95%* CI	*P* value
Age (years)	1.04	(1.03, 1.04)	< 0.001	1.04	(1.03, 1.04)	< 0.001
Square root of age	1.72	(1.60, 1.84)	< 0.001			
Age less than 65 indicator	0.33	(0.27, 0.41)	< 0.001			
Index Diagnosis vs. Bipolar Disorder						
Depressive Disorder	0.95	(0.78, 1.14)	0.57	1.02	(0.79, 1.32)	0.88
Schizophrenia	1.96	(1.67, 2.29)	< 0.001	1.99	(1.57, 2.53)	< 0.001
Generation of Index AP vs. First generation						
Multiple	0.88	(0.58, 1.33)	0.54	0.80	(0.52, 1.21)	0.29
Second generation	0.72	(0.59, 0.87)	< 0.001	0.85	(0.70, 1.03)	0.09
Dose (continuous effect for dose≤100 mg/day of chlorpromazine)	1.91	(1.38, 2.66)	< 0.001	1.65	(1.17, 2.31)	< 0.01
Dose (continuous effect for dose> 100 mg/day of chlorpromazine)	1.05	(1.02, 1.08)	< 0.01			
CCI	1.06	(1.00, 1.12)	< 0.05			
Depressive disorders	0.78	(0.68, 0.89)	< 0.001			
Bipolar and related disorders	0.84	(0.72, 0.97)	< 0.05	1.39	(1.11, 1.75)	< 0.01
Dementia	2.04	(1.38, 3.01)	< 0.001			
Diabetes	1.52	(1.29, 1.79)	< 0.001	1.13	(0.96, 1.34)	0.14
Number of EPS	1.91	(1.60, 2.29)	< 0.001			
History of EPS	2.37	(1.88, 2.99)	< 0.001	1.35	(0.74, 2.47)	0.33
Parkinsonism	4.29	(1.38, 13.33)	< 0.05	1.43	(0.44, 4.72)	0.55
Bradykinesia	2.48	(1.92, 3.21)	< 0.001	1.44	(0.77, 2.68)	0.25
Tremors	3.93	(1.96, 7.89)	< 0.001	2.12	(0.97, 4.60)	0.06
Myoclonus	4.27	(1.07, 17.11)	< 0.05	2.33	(0.56, 9.7)	0.25

*AP* antipsychotic, *CCI* Charlson Comorbidity Index, *CI* confidence interval, *EPS* extrapyramidal symptoms, *LASSO* least absolute shrinkage and selection operator

**Fig. 1:**
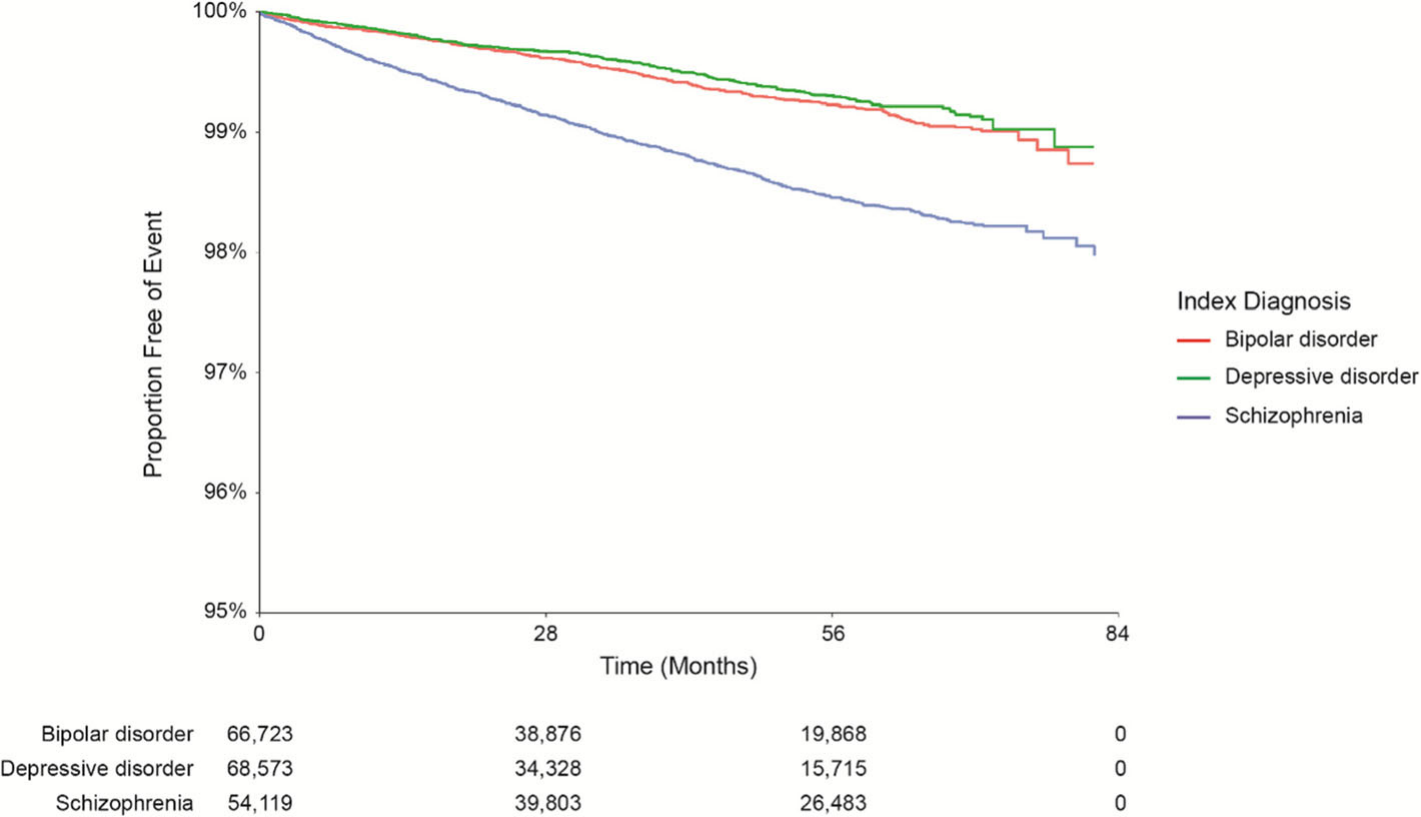
Kaplan-Meier curves of time to TD diagnosis. Estimated TD incidence rate within 7 years after antipsychotic drug initiation were stratified by index psychiatric disorder diagnosis. TD, tardive dyskinesia

Kaplan–Meier (KM) curves of time to TD diagnosis stratified by various risk factors were also generated. Consistent with the univariate Cox results, the time to TD diagnosis was shorter in patients with schizophrenia than in those with bipolar or depressive disorder (Fig. 1**)**. In addition, the time to TD diagnosis was shorter in patients with a history of EPS than in those without, and longer in patients taking second-generation antipsychotics than in those taking first-generation or multiple first- and second-generation antipsychotics (data not shown).

### TD prediction models

**Fig. 2:**
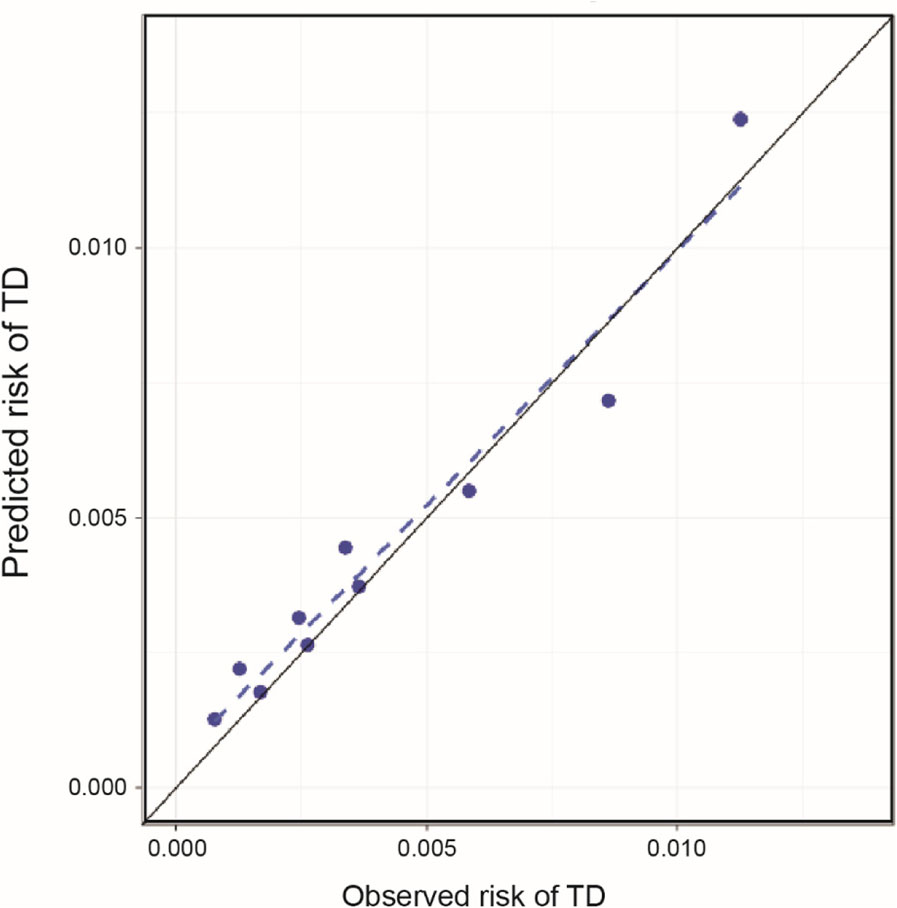
Calibration plot for the re-estimated LASSO prediction model. A least absolute shrinkage and selection operator (LASSO) prediction model was used to identify risk factors for TD. The model was developed with data in the modeling set and validated and re-estimated with the validation data set. The risk of TD at 2 years after the index date as predicted by the model was compared with actual TD observed, within the validation set (one-third of the data set). Concordance was 70.6%, Hosmer–Lemeshow goodness-of-fit test, *P* = 0.32. TD, tardive dyskinesia

A multivariate Cox prediction model was estimated using the predictors selected by the LASSO. The resulting prediction model (“re-estimated LASSO model”) had a clinically meaningful concordance of 70.6% and was well calibrated (*P* = 0.32 for Hosmer–Lemeshow goodness-of-fit test) (Fig. 2). The multivariate model selected and estimated by the LASSO had similar predictive performance (concordance = 70.5%, *P* = 0.46 for Hosmer–Lemeshow goodness-of-fit test) and covariate estimates. In the re-estimated LASSO model, age (HR = 1.04, *P* < 0.001), diagnosis of schizophrenia (HR = 1.99, *P* < 0.001, compared with bipolar), dosage of antipsychotic medication (up to 100 mg/day of chlorpromazine equivalent, HR = 1.65, *P* < 0.01), and presence of bipolar and related disorders (HR = 1.39, *P* < 0.01) were significantly associated with an increased risk of TD. Other potential predictors of TD diagnosis included history of EPS, movement disorders (parkinsonism, bradykinesia, tremors, and myoclonus), and diabetes (Table 3). The use of second-generation antipsychotic medication was associated with a modest reduction in risk of TD (HR = 0.85; Table 3).

## Discussion

The variability observed in the onset, developmental pattern, and response to interventional treatment make TD a difficult condition to diagnose and to treat [3, 5, 9]. Because TD is sometimes irreversible, early detection and prevention of TD in patients with high-risk status is an important strategy for the clinical management of TD [3, 11, 21].

Despite recent advances, identification of TD predictors remains challenging for researchers and clinicians [1]. There are common methodological confounding factors and considerable study limitations, including that TD can mimic signs of the underlying comorbidity or that it can be masked by antipsychotics [1, 3, 4, 19, 21]. In the current study, the use of large claims data provided real-world evidence for the incidence of TD due to antipsychotic medication use among patients with schizophrenia, major depressive disorder, or bipolar disorder. Furthermore, the analytical approach was designed to help identify risk factors for TD by examining their associations with TD diagnosis both in isolation and in combination with a large set of factors via multivariate modeling, which, to our knowledge, had not previously been developed or validated in US populations. Consistent with prior studies [7, 12–15], of the baseline and index-date characteristics under consideration, patient age, diagnosis of schizophrenia, dosage of antipsychotic medication (up to 100 mg/day of chlorpromazine equivalent), and presence of bipolar and related disorders were associated with greater risk of TD in patients taking antipsychotics. Interestingly, the presence of bipolar and related disorders was found to be associated with a significant decrease in the risk of TD in the univariate analyses, but this association was reversed in the multivariate model, indicating the importance of examining these associations while accounting for other factors. Also, female sex, a variable previously observed to be associated with an increased risk of TD [11, 29], was not among the best predictors of TD in this study.

Although the relationship we found between predictors included in this study and TD diagnosis was associative rather than causal in nature, these observations are clinically relevant findings that can aid in risk-mitigation planning and implementation. The resulting prediction model can provide the risk or probability that TD will occur within any time period after the index date (e.g. 1 or 2 years) for each patient based on their baseline or index-date prognostic factors, which can guide decision-making regarding treatment and follow-up management from the time of the diagnosis of the psychiatric disorder.

There has been considerable debate regarding the attrition in TD incidence since widespread adoption of second-generation antipsychotics. One study previously reported a point prevalence of 13% with second-generation antipsychotics versus 32% with first-generation, whereas other studies have reported no differences [19, 30–32]. In addition, multiple studies have challenged the notion that second-generation antipsychotics are relatively free of the risk of TD [19, 20]. The current study utilized univariate and multivariate Cox models to re-assess the comparative risk of TD associated with both drug classes. Compared with first-generation antipsychotics, the use of second-generation antipsychotics was associated with a statistically significant reduction in the risk of TD when analyzed using a univariate Cox model. However, this reduction was more modest and no longer significant in the final LASSO prediction model.

### Limitations

Although the study yielded a well-calibrated prediction model with a clinically meaningful concordance of 71%, it was subject to limitations that are inherent to using a claims database. The study population was limited to patients within the Medicaid database and represented only six US states, and therefore its findings may not be generalizable to other patient populations. TD was relatively rare in this study population (the KM-estimated proportion of patients with TD at 7 years after antipsychotic drug initiation was less than 2%), which is partly due to a relatively short follow-up period for the condition under study. As a result, the prediction performance of the model, in terms of its discrimination power (concordance), was acceptable rather than excellent. This issue was mitigated by using the LASSO methodology, which can provide better predictions than standard regression by avoiding overfitting in data sets with few events. In addition, comorbidities may have been underestimated because they were identified using diagnosis codes, which are typically used for administrative purposes. Although the data set used in this analysis provides a large and representative real-world evidence of patients in the US, it spans a limited follow-up time (up to 7 years), which is an important limitation given that the development of TD is associated with long-term use of antipsychotics. Thus, the rate of TD claims in these data was low compared with the prevalence of TD, which is 20–50% among all patients treated with antipsychotics [9]. Another likely limitation of the study is that, due to its observational design, results may have been confounded due to unobserved factors that cannot be accounted for in multivariable regression analyses. For example, this study did not examine the role of race/ethnicity in the risk of TD, which was previously identified as a potential risk factor [13]. Also, given the large number of antipsychotic medications and the relatively low number of TD events observed in these data, the risk of TD was analyzed by class of antipsychotics and not by each antipsychotic separately. Thus, the risk of TD associated with each specific antipsychotic was not ascertained in this analysis.

The paucity of claims for TD in the database, in comparison with the reported prevalence for motor disorder of up to 40% reported previously [4], may affect the prognostic implications of the findings reported herein. One possibility is that the constraints of a retrospective study design may lead to underestimation of TD prevalence [33]. However, the discrepancy between observed and anticipated TD prevalence rates in the study may underscore a more-systemic problem regarding the epidemiology of TD, namely the potential underreporting due to a lack of clinical awareness or standardization of diagnostic criteria [34].

## Conclusions

This study identified a group of factors associated with the development of TD among patients who had psychiatric disorders treated with antipsychotics. The prediction model developed and validated herein can help physicians identify patients at high risk for TD in order to develop treatment and monitoring plans that help patients maintain their quality of life.

## Additional files

Additional file 1: ICD-9-CM and ICD-10 CM codes for selected comorbidities and GPIs for antipsychotics. (DOCX 37 kb)

Additional file 2: Sample selection flow chart. (DOCX 635 kb)

## Data Availability

The data that support the findings of this study are available from Analysis Group, but restrictions apply to the availability of these data, which were used under license for the current study, and so are not publicly available. Data are, however, available from the authors upon reasonable request and with permission of Analysis Group.

## Abbreviations

CCI: Charlson Comorbidity Index
EPS: Extrapyramidal symptoms
GPI: Generic Product Indicator
HR: Hazard ratio
ICD-10-CM: International Classification of Diseases, Tenth Revision, Clinical Modification
ICD-9-CM: International Classification of Diseases, Ninth Revision, Clinical Modification
KM: Kaplan–Meier
LASSO: Least absolute shrinkage and selection operator
TD: Tardive dyskinesia
