# Supplementary material for: Risk assessment and prediction of TD incidence in psychiatric patients taking concomitant antipsychotics: a retrospective data analysis
Source: BMC Neurol. 2019 Jul 20;19:174. doi: 10.1186/s12883-019-1385-4 (PMC6642740; doi:10.1186/s12883-019-1385-4)
Supplement: Supplementary file 1 — ICD-9-CM and ICD-10 CM codes for selected comorbidities and GPIs for antipsychotics. (DOCX 37 kb) [file 12883_2019_1385_MOESM1_ESM.docx]

**ICD-9-CM and ICD-10 CM Codes for Selected Comorbidities and GPIs for Antipsychotics**

| Comorbidities | ICD-9-CM Diagnosis Codes | ICD-10-CM Diagnosis Codes |
| --- | --- | --- |
| Schizophrenia spectrum and other psychotic disorders (excluding schizophrenia) | 293.81, 293.82, 293.89, 297.1, 298.8, 298.9, 301.22 | F06.2, F15.951, F06.1, F21, F22, F23, F29, F06.0, F60.1 |
| Substance-related and addictive disorders | 291.81, 291.9, 292.0, 292.89, 292.9, 303.00, 303.90, 304.00, 304.10, 304.20, 304.30, 304.40, 304.50, 304.60, 304.90, 305.00, 305.1, 305.20, 305.30, 305.40, 305.50, 305.60, 305.70, 305.90 | F10, F11, F12, F13, F14, F15, F16, F17, F18, F19, Z71.41, Z71.51 |
| Depressive disorders | 296.2, 296.3, 300.4, 625.4, 311 | F34.1, F33, F32 |
| Bipolar and related disorders | 293.83, 296.4, 296.5, 296.7, 296.80, 296.89, 301.13 | F06.30, F10.14, F10.24, F10.94,  F13.94, F14.24, F15.14, F15.94,  F16.24, F30, F31, F34, F39 |
| Trauma- and stressor-related disorders | 308.3, 309.81, 309.0, 309.24, 309.28, 309.3, 309.4, 309.9, 309.89, 313.89 | F43, R45.7 |
| Anxiety disorders | 309.21, 312.23, 300.29, 300.23, 300.01, 300.22, 300.02, 293.84, 300.09, 300.00 | F06.4, F40, F41, F93.0 |
| Sleep-wake disorders | 307.45, 307.46, 307.47, 327.21, 327.23, 327.24, 327.25, 327.26, 327.42, 333.94, 347.00, 347.01, 347.10, 780.52, 780.54, 780.57, 780.59, 786.04 | F51, G47, G25.81, R06.3 |
| Personality disorders | 301.0, 301.1, 301.20, 301.4, 301.5, 301.6, 301.81, 301.82, 301.83, 301.89, 301.9 | F60, F34.0, F34.1 |
| Unspecified extrapyramidal symptoms | 333.9 | G25.9 |
| Parkinsonism | 332.1 | G25.11 |
| Tremor | 333.1 | G25.1 |
| Myoclonus | 333.2 | G25.3 |
| Tics of organic origin | 333.3 | G25.6 |
| Acute dystonia due to drugs | 333.72 | G24.02 |
| Neuroleptic malignant syndrome | 333.92 | G21.0 |
| Akathisia | 333.99 | G25.71 |
| Bradykinesia | 781 | G25.8 |
| Down syndrome | 758.0 | Q90 |
| Autism | 299.0 | F84.0 |
| Dyslexia and other scholastic disorders | 315.0, 784.6 | R48, F81 |
| Traumatic brain injury | 348.5, 851, 852, 853, 907.0 | S06.1, S06.2, S06.5, S06.6 |
| Brain damage | 348.1, 348.4, 854 | G93.1, G93.5, G93.8, S06.1-S06.3, S06.9 |
| Diabetes (with and without chronic complications) | 250.0-250.3, 250.8, 250.9, 250.4-250.7 | E100-E101, E106, E108-E111, E116, E118-E121, E126, E128-E131, E136, E138-E141, E146, E148, E149, E102-E105, E107, E112-E115, E117, E122-E125, E127, E132-E135, E137, E142-E145, E147 |
| Alcohol abuse | 305.0, 303, V65.42, V11.3 | F10.1, F10.2. Z71.41 |
| Smoking history | 305.1, V15.82, V65.42 | F17.21, F17.29, Z53.01, Z71.6, Z72.0 |
| **First-generation antipsychotics** | | |
|  | Generic name | GPI |
|  | Acetophenazine | 5920001010 |
|  | Carbamazepine | 5920001310 |
|  | Chlorpromazine | 5920001500, 5920001510 |
|  | Chlorprothixene | 5930001000, 5930001020 |
|  | Fluphenazine | 5920002510 |
|  | Haloperidol | 5910001010, 5910001020 |
|  | Loxapine | 5915402000, 5915402010, 5915402020 |
|  | Molindone | 5916005010 |
|  | Perphenazine | 5920004500 |
|  | Prochlorperazine | 5920005500 |
|  | Promazine | 5920006010, 9672764370 |
|  | Thioridazine | 5920008000, 5920008010 |
|  | Thiothixene | 5930002010, 5930002020 |
|  | Trifluoperazine | 5920008510 |
| **Second-generation antipsychotics** | | |
|  | Aripiprazole | 5925001500 |
|  | Asenapine | 5915501510 |
|  | Brexpiprazole | 5925002000 |
|  | Cariprazine | 5940001810 |
|  | Clozapine | 5915202000 |
|  | Iloperidone | 5907003500 |
|  | Lurasidone | 5940002310 |
|  | Olanzapine | 5915706000 |
|  | Paliperidone | 5907005000 |
|  | Pimavanserin | 5940002820 |
|  | Quetiapine | 5915307010 |
|  | Risperidone | 5907007000 |
|  | Ziprasidone | 5940008510 |
| Other | | |
|  | Lithium | 5950001000, 5950001010, 5950001020 |

GPI, Generic Product Indicator; ICD-9-CM, International Classification of Diseases, Ninth Revision, Clinical Modification; ICD-10-CM, International Classification of Diseases, Tenth Revision, Clinical Modification.
