# Supplementary material for: Risk assessment and prediction of TD incidence in psychiatric patients taking concomitant antipsychotics: a retrospective data analysis
Source: BMC Neurol. 2019 Jul 20;19:174. doi: 10.1186/s12883-019-1385-4 (PMC6642740; doi:10.1186/s12883-019-1385-4)
Supplement: Supplementary file 2 — Sample selection flow chart. (DOCX 635 kb) [file 12883_2019_1385_MOESM2_ESM.docx]

**Sample Selection Flow Chart**

Patients’ data were collected from a Medicaid database, and those who met the selection criteria were included in the analyses. TD, tardive dyskinesia.

**
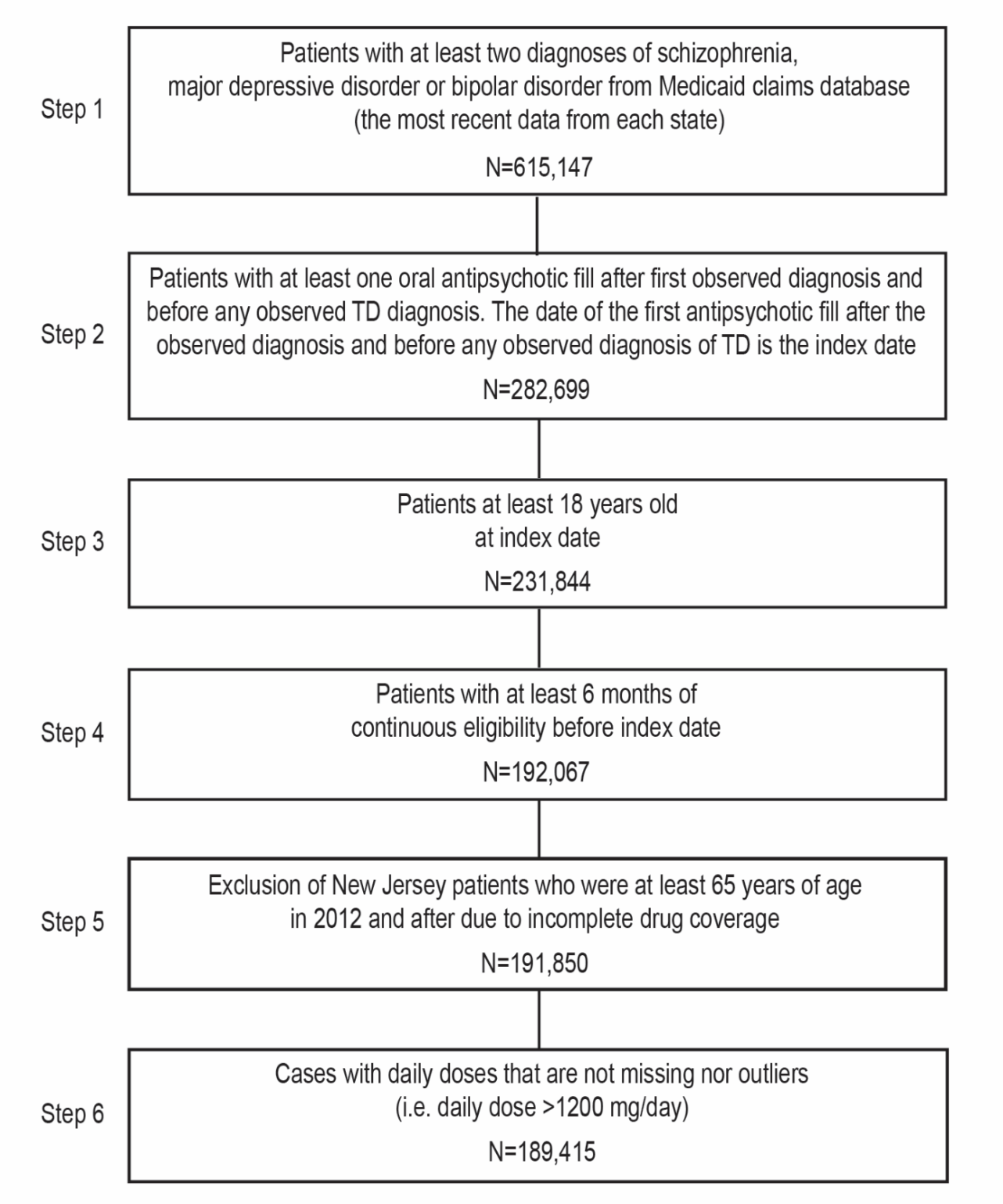
**
